# Factors contributing to high performance of sows in free farrowing systems

**DOI:** 10.1186/s40813-024-00366-w

**Published:** 2024-05-02

**Authors:** Emma M. Baxter, Nicola Bowers, Rebecca King, Sarah Brocklehurst, Sandra A. Edwards

**Affiliations:** 1 Animal and Veterinary Sciences Research Group, Scotland‘s Rural College (SRUC), West Mains Road, Edinburgh, EH9 3JG UK; 2 Farmvet Integrated Livestock Services, Unit 3 Zenith Park Network Centre, Whaley Road, Barnsley, S75 1HT UK; 3 ADAS Leeds, 4205 Park Approach, Leeds, LS15 8GB UK; 4 Biomathematics and Statistics Scotland (BioSS), Edinburgh, EH9 3FD UK; 5 School of Natural and Environmental Sciences, Newcastle University, Newcastle upon Tyne, NE1 7RU UK

## Abstract

**Background:**

Pressure to abolish farrowing crates is increasing, and producers are faced with decisions about which alternative system to adopt. For sow welfare, well designed free farrowing systems without close confinement are considered optimal but producers have concerns about increased piglet mortality, particularly crushing by the sow. Reporting accurate performance figures from commercial farms newly operating such systems could inform the transition process. This study investigated performance on three commercial farms operating four different zero-confinement systems, three of which were newly installed. A total of 3212 litters from 2920 sows were followed from farrowing to weaning over a three-year period with key performance indicators (KPIs) recorded. Mixed Models (LMMs, GLMMs) determined the influence of different factors (e.g. farrowing system, sow parity, management aspects) and litter characteristics on performance, including levels and causes of piglet mortality.

**Results:**

Piglet mortality was significantly influenced by farm/system. Live-born mortality ranged from 10.3 to 20.6% with stillbirths ranging from 2.5 to 5.9%. A larger litter size and higher parity resulted in higher levels of mortality regardless of system. In all systems, crushing was the main cause of piglet mortality (59%), but 31% of sows did not crush any piglets, whilst 26% crushed only one piglet and the remaining sows (43%) crushed two or more piglets. System significantly influenced crushing as a percentage of all deaths, with the system with the smallest spatial footprint (m^2^) compared to the other systems, recording the highest levels of crushing. Time from the start of the study influenced mortality, with significant reductions in crushing mortality (by ~ 4%) over the course of the three-year study. There was a highly significant effect of length of time (days) between moving sows into the farrowing accommodation and sows farrowing on piglet mortality (*P* < 0.001). The less time between sows moving in and farrowing, the higher the levels of piglet mortality, with ~ 3% increase in total mortality every five days. System effects were highly significant after adjusting for parity, litter size, and days pre-farrowing.

**Conclusion:**

These results from commercial farms demonstrate that even sows that have not been specifically selected for free farrowing are able, in many cases, to perform well in these zero-confinement systems, but that a period of adaptation is to be expected for overall farm performance. There are performance differences between the farms/systems which can be attributed to individual farm/system characteristics (e.g. pen design and management, staff expertise, pig genotypes, etc.). Higher parity sows and those producing very large litters provide a greater challenge to piglet mortality in these free farrowing systems (just as they do in crate systems). Management significantly influences performance, and ensuring sows have plenty of time to acclimatise between moving in to farrowing accommodation and giving birth is a critical aspect of improving piglet survival.

**Supplementary Information:**

The online version contains supplementary material available at 10.1186/s40813-024-00366-w.

## Background

Pressure to abolish farrowing crates is increasing, particularly in Europe, where political debate about the continued use of close-confinement systems has intensified following successful campaigns by animal advocacy groups to ‘End the Cage Age’ [1]. The result of this European Citizens’ Initiative (ECI) was a commitment by the European Commission (first tabled in 2021) to phase out and eventually prohibit the use of close-confinement systems for all farmed species. There was also a realisation by many in the industry that transitioning to higher welfare alternatives with less confinement is inevitable and ‘free farrowing is a matter of how not when’ [2]. However, barriers to uptake of such systems do exist, including costs, the potential for increased labour demand and continued concerns about increases in piglet mortality, particularly from crushing by the sow. Some farmers have expressed greater confidence in managing piglet mortality in alternative farrowing systems [2] and good performance results are reported in some countries already operating free farrowing, notably Norway and Switzerland (e.g. [3–6]). Many of those transitioning to alternative systems in countries where there are currently no restrictions on the use of confinement for farrowing and lactation have favoured adopting temporary crating systems as an ‘insurance policy’ to lessen this concern over potential increases to piglet mortality [7]. Whilst good temporary crating systems offer better sow welfare than conventional crates [7], they do not confer the same welfare benefits as true free farrowing systems (i.e. zero-confinement) due to the restriction of highly motivated prepartum nest-site seeking and nest-building behaviours, which are characterised by increased activity, rooting, pawing, searching for suitable nest-building materials, turning around and arranging those materials [8–10]. Feedback from building and completing a nest pre-partum can affect neuroendocrine regulation of maternal behaviour during and post-partum [11], with evidence of improvements in sow hormonal balance and colostrum quality [12], as well as positive maternal behaviours including increased responsiveness to piglet distress calls, increased maternal bonding and a positive effect on nursing behaviour [9, 13–15]. Reduced piglet birth intervals, reduced stillbirths and reduced postnatal mortality have also been reported (reviewed by [16]). Farmers installing temporary crating systems also risk incurring the same public reaction as poultry farmers who installed ‘enriched cages’ to replace battery cages for laying hens; the consumer’s view was that ‘a cage is still a cage’ [17, 18]. The installation of enriched cages for laying hens proved costly as such systems are precluded from many markets and are also targeted under the ECI [1].

Despite the concerns of producers regarding potential increases in piglet mortality when the sow is loose for farrowing, the consumer’s negative view on confinement, particularly for expectant mothers, is global and enduring [19–21]. An EU-wide survey on the attitudes of European citizens to animal welfare in 2023 [21] reported that 89% of respondents believe that ‘ensuring animals are not kept in individual cages is important’.

There has been a great deal of research into designing zero-confinement farrowing systems that optimise animal welfare [8, 22] and data have been published demonstrating that some systems can offer equivalent levels of piglet mortality to conventional farrowing crates [23, 24]. However, it is likely that farmers will be more receptive to data that are commercially relevant to them, coming from free farrowing systems having been adopted or trialled (i.e. partial adoption [25]) on commercial farms. However, data on performance from commercial farms, specifically operating zero-confinement systems, are sparce. Generally, these types of data are only available from countries that have been operating free farrowing systems for a sustained period of time (e.g. Sweden 1987; Norway 2000; Switzerland 2007 (after a 10-year transition period)) and, whilst these countries offer valuable insights about their experience, the performance data may not be as valuable to someone considering transitioning now.

This project aimed to address this knowledge gap by collecting key performance indicators from sows farrowing in different zero-confinement systems operated on commercial UK farms. Our objective was to report commercially relevant performance data, determining any influential biological and environmental factors affecting performance in these zero-confinement systems, particularly piglet mortality.

## Methods

### Animal housing and management

The study was conducted over a three-year period (2013–2016) on three participating commercial farms in the United Kingdom, operating four different free farrowing (FF) systems as well as conventional farrowing crates. The project was focused on the FF systems and did not involve comparative work in crates. Data were collected on 3212 litters from 2920 (primi- and multi- parous) sows. Table 1 describes the farms, farrowing systems, sow genetics and breeding herd management. Pictures of systems are provided in Supplementary Materials (Figure S1).

**Table 1:** Farm and system descriptions

	FARM 1	FARM 2	FARM 3	
Herd characteristics	1700 JSR-90 breeding sows and gilts (JSR Genetics, Driffield, UK) bred with various sire lines (JSR Genetics 900, Rattlerow Farms Ltd (Suffolk, UK) Danish Duroc, Elite Sires (Coleraine, NI))	750 Large White x Landrace breeding sows and gilts (PIC, Nantwich, UK), bred with a Hampshire sire line (Hermitage PIC, Kilkenny, Ireland).	1300 Camborough breeding gilts and sows (Genus PIC, Basingstoke, UK), bred with Hampshire semen collected on-site for artificial insemination.	
Gestation housing	Stable groups (*n* = 75–82 sows) according to gestation stage (gilts in separate groups). Housed in naturally ventilated barns with deep straw bedding.	Dynamic groups (*n* = 300 parities 2–3 and *n* = 400 parities 4+). Housed in large naturally ventilated barns with deep straw bedding. Gilts were housed in naturally ventilated straw-bedded pens in stable groups (*n* = 16–20) according to their farrowing date.	Stable groups (*n* = 12–16 per pen), in naturally ventilated straw-bedded pens. Grouped according to age, for gilts, or by size for multiparous sows.	
Farrowing System	**A**	**B**	**C**	**D**
	One room with 72 freedom farrowing pens (3.0 m x 2.0 m, 75% solid concrete, 25% slatted flooring - Fig. 1A). Pens are zero-confinement. Manufacturers = Jyden Bur A/S (now SKIOLD GROUP, Denmark)	Two rooms with 8 PigSAFE pens (3.7 m x 2.4 m, 40% solid with slots, 60% slatted flooring), 4 with feeding stall in one room (Fig. 1B) and 4 without feeding stall in one room. Pens are zero-confinement. Manufacturers = Quality Equipment, UK and A.M.Warkup UK – built according to PigSAFE design specifications detailed at www.freefarrowing.org	Eight rooms with 168 360° Freedom Farrowers® (between 16–20 places per room) System with temporary crating possibility on a fully slatted plastic floor (2.50 m x 1.80 m pen with a 2.50 m x 0.50 m stainless steel crate which measured 2.50 m x 1.60 m at sow shoulder height when opened) (Fig. 1C). Crates operated permanently in the open position for trial. Manufacturers = Midland Pig Producers, UK	62 kennel and run (‘Solari’) pens in rows of individual units with an inside area (2.30 m x 1.20 m solid floor) and an outdoor run (2.55 m x 2.05 m) (Fig. 1D). Pens are zero-confinement. System D was a long established system constructed from timber and brick in the 1960s.

Sows were assigned to farrowing accommodation within each farm based on the farm’s routine schedule (i.e. not experimentally controlled). Animals were generally moved into the farrowing accommodation approximately four days before their expected farrowing date (average 4.17 days ± SD 2.42).

### Substrate provision and heating

**Fig. 1:**
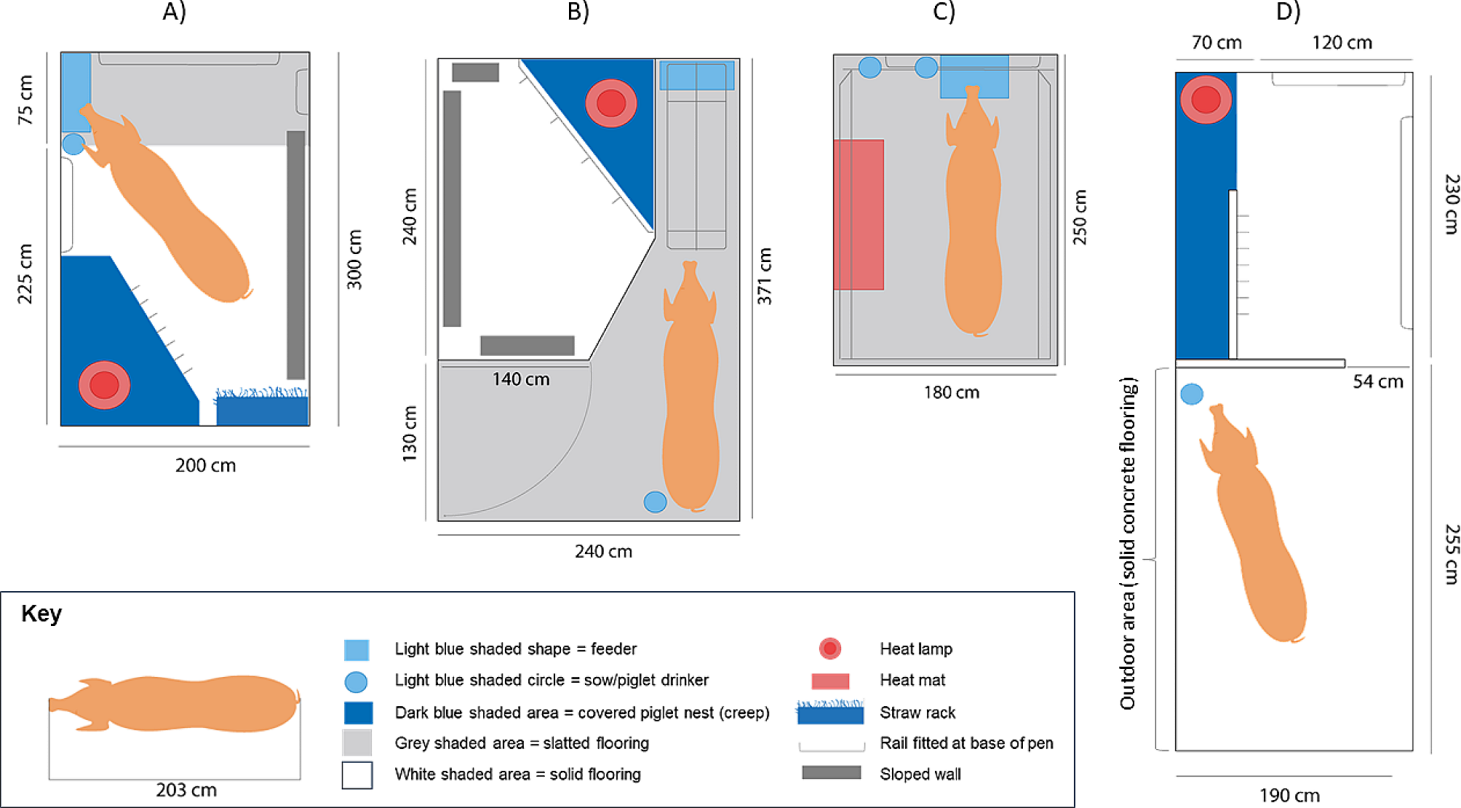
Free farrowing systems operated on three commercial farms. Farm 1 operated system (**A**) (a Danish Free Farrower design), Farm 2 operated system (**B**) (PigSAFE design) and Farm 3 operated both systems (**C**) (a ‘360®’ temporary crate in its open position for the trial) and (**D**) (a ‘Solari’ system with an indoor kennel and outdoor run). All drawings to scale and created by Pig 333 from author description. Pig length represents the 95th percentile of a modern hyperprolific sow as determined by Nielsen et al. 2018 [26]

Upon entry to farrowing accommodation on Farm 1, in system A (Fig. 1A) wood shavings and 5 kg long-stemmed straw (i.e. enough to cover the solid floor area) was provided in the nesting area and shavings and straw provided in the creep area. The building containing the farrowing pens was initially kept at 22 ± 1 °C and was gradually reduced automatically to 18 ± 1 °C. A 75w heat lamp was suspended over the covered creep area 1 m above the creep floor. Pre-partum, pens were cleaned out daily with soiled straw and shavings being removed and replenished as necessary. Pens were cleaned out weekly post-partum to remove any soiled bedding and a dry powder applied to the solid area to keep it dry and avoid slipping.

On Farm 2, in system B (Fig. 1B) 10 kg long-stemmed straw was provided to cover the nesting area when sows were moved in. The building containing the farrowing pens was initially kept at 22 °C and was gradually reduced automatically to 18 °C. A 75w heat lamp was suspended over the covered creep area 1 m above the creep floor and the creep area also contained a heat pad starting at 35 °C and reducing to 30 °C by weaning. Pre-partum, pens were cleaned out daily with soiled straw being removed and replenished as necessary. Post-partum, soiled straw was removed daily until no straw was present in the pen.

On Farm 3, in system C (Fig. 1C) stockpeople gave approximately two handfuls of shredded paper when farrowing was imminent and the buildings containing the farrowing pens were kept at 22 °C, with the additional heat mat along one side of each pen starting at 36 °C and reducing to 30 °C by weaning. Room temperature was gradually reduced automatically to 18 °C by day ten post-partum and to 16 °C by weaning. In system D (Fig. 1D) the nest area contained 5 kg of long-stemmed straw (i.e. enough to cover the solid floor) from the day of sow entry into the farrowing system, whilst the entire creep floor was covered in wood shavings. Pens on system D were routinely cleaned out weekly with straw and wood shavings replenished. Pre-partum, additional straw or wood shavings were added to nests when required and soiled straw was removed and replenished post-partum. As the pens had no ambient temperature controls, a 400 w electric heater was located at one end of each covered creep, these being individually switched off three to five days post-partum.

### Sow and piglet husbandry

#### Feeding

On Farm 1, gestating sows were fed a wet ration of approximately 2.5-3 kg per day (14.47% CP, 13.11 DE MJ/Kg) through electronic sow feed stations (SCHAUER Agrotronic GmbH, Austria) allowing individual feeding. Water was available ad libitum. Upon entry into the farrowing accommodation, sows were fed on an automated liquid feed system once a day into troughs over the slatted area of the pen. Sows were fed a transition ration (18.32% CP, 14.07 DE MJ/Kg) pre-farrowing and for one week post-farrowing and then changed onto a lactation ration (19.77% CP, 14.39 DE MJ/Kg) on an automatic feed curve to gradually increase feed throughout lactation. Once all sows had farrowed within the building, feeding frequency was increased to twice daily and then increased to four times daily by the end of lactation. Water was available ad libitum for sows (bite drinker) and piglets (bowl and nipple drinker) (Fig. 1A). Creep feed (Primary Diets, AB Agri Ltd, Peterborough, UK) was introduced in the creep area from 10 days of age and increased according to appetite until weaning.

On Farm 2, gestating sows were fed a pelleted gestation diet (gilts: 12.52% CP, 12.08 DE MJ/kg; sows: 11.95 CP%, 12.33 DE MJ/kg) through electronic sow feed stations allowing individual feeding (Nedap Livestock Management, The Netherlands) with levels based on a feed curve set by body condition, time of year and stage of gestation. Gilts were fed as a group using dump feeders in their accommodation (Table 1). Water was available ad libitum. Upon entry into the farrowing house, sows had pelleted lactation feed (17% CP 13.36 DE MJ/kg) dispensed through a Gestal feed dispenser (Jyga Technologies, Canada) set to a curve, which could be increased during lactation and adapted for individuals depending on size of litter, parity and appetite. Feed increased from 1.5 kg pre-farrowing to up to 8-12 kg during lactation. Creep feed for piglets (Primary Diets, AB Agri Ltd, Peterborough, UK) was introduced in the creep area from 10 days of age and increased according to appetite until weaning. Water was available ad libitum for all sows (bite drinker) and piglets (nipple drinker) (Fig. 1B).

On Farm 3, gestating sows were fed via dump-feeders once daily with approximately 3 kg of pelleted feed per sow per day (gilts = 12.42% CP, 12.52 DE MJ/Kg; sows = 11.85% CP, 12.47 DE MJ/Kg). Water was available ad libitum. Upon entry into the farrowing accommodation, sows were hand-fed once daily in the morning, onto the floor of the nest area in system D or troughs in system C, until all sows in a building had farrowed, after which sows were fed twice a day. Feed was a lactation diet (15.98% CP, 13.69 DE MJ/kg) and was gradually increased from 2 to 10 kg per sow per day throughout lactation. A handful of creep feed (Primary Diets, AB Agri Ltd, Peterborough, UK; followed by Flat Deck, A-One Feed Supplements Ltd, Thirsk, UK) was provided once daily on the floor in all systems from approx. 10 days of age until weaning. Water was available ad libitum for sows (button drinker – System C) and piglets (nipple drinker – System C– Fig. 1C) and from a water bowl shared by sows and piglets for System D (Fig. 1D).

#### Piglet procedures

At the time of the study, in accordance with veterinary advice for each farm and in accordance with regulations (The Welfare of Farmed Animals (England) Regulations, 2007) permitting certain procedures once ‘measures to improve environmental conditions or management systems have first been carried out’, piglets were tail docked on Farms 2 and 3 and teeth were clipped or ground. None of these procedures were implemented on Farm 1. Piglets were given 1 ml of Uniferon (Farm 1) (Pharmacosmos A/S, Holbaek, Denmark; each ml contains 200 mg iron(III) as iron(III) hydroxide) or Gleptosil (Farms 2 and 3) (Ceva Animal Health Ltd, Amersham, UK; each ml contains 200 mg iron as gleptoferron complex (498 mg/ml)). On Farms 2 and 3 piglets were also given 0.5 ml of Betamox (Norbrook Laboratories Ltd, Newry, Northern Ireland; each ml contains 150 mg (15% w/v) Amoxicillin (as Amoxicillin Trihydrate 17.21%w/v)) within 24 h of birth.

## Data collection

Data were collected on sows from the time that they were moved into the farrowing accommodation until weaning. Days were assigned a number based on the farrowing date (i.e. farrowing date = day 0 (D0), day before farrowing = day − 1 (D-1) etc.). On move-in day, sows were weighed (on farms where weighing equipment was available) and body condition scored (1–5 scale of increasing body condition, visually assessed using the AHDB scoring system^1^). Sows were scored for signs of lameness according to D’Eath’s protocol [27]. On D0, whether or not a sow was induced to farrow, savaged any piglets, was given oxytocin (Oxytocin-S, 10 iu/ml, single dosage 0.2-1.0 ml) in the case of dystocia or post-birth milking difficulties, and/or an internal examination because of farrowing problems was recorded. The number of functional teats were counted. The key performance indicators (KPIs) of total litter size, number born dead, alive and mummified were recorded along with litter birth weight (on farms where weighing equipment was available). Any other deaths or injuries were noted, and the cause of death was ascertained by gross examination of piglets using the descriptions in Table 2. Fostering information was recorded throughout lactation in order to calculate total mortality and live-born mortality at weaning adjusting the number of piglets at risk for numbers fostered on/off. At weaning (average weaning age 25.7 ± SD 4.13 days) sows were weighed (where possible), scored for signs of lameness and body condition scored. The number of piglets weaned per sow, and litter weaned weight (where possible) were recorded.

**Table 2:** Causes of death of piglets and their descriptions

Cause of death	Definition
Stillborn	Fully formed piglet not breathing at birth (if present for births), periople still present on hooves, could be found in placental fluids.
Crushed	Piglet found squashed or bruised in appearance or with broken bones.
Low viability	Piglet small and may have features of intrauterine growth retardation – domed head, wrinkles around mouth, bulging eyes.
Starved	Piglet very thin, dehydrated skin with pin bones and individual ribs visible, vertebrae prominent.
Other	Scour, blind anus, deformations, disease and any unidentified causes. Often euthanized.

## Data processing and statistical analyses

Data were processed to calculate per litter percentage total mortality (TM%), percentage live-born mortality (LBM%) and percentage still-born mortality (SB%) for the period from birth to weaning (see data dictionary in Supplementary Materials Table S1 which lists numeric data collected and equations for all derived numeric variables). Fostering adjustments were taken into account. Mortality results were also split between any deaths that occurred pre-processing (PRE) (i.e. within the first 24 h before stockpeople undertook any interventions and piglet husbandry procedures) and post-processing to weaning (POST). This was done to distinguish effects that could more easily be attributed to the sow without the influence of human intervention, including fostering adjustments. The percentages of each type of live-born death (Crushed (CSH), Low viability (LV), Starved (STV), and Other) were calculated similarly for the period from birth to weaning, and split to distinguish PRE and POST processing deaths (see Supplementary Materials Table S1). Analyses focused on each type of live-born death as the percentage of litter size (adjusting for fostering as appropriate, referred to in data dictionary as Crushed% etc.), but some results are also given on the percentage of the death types within all deaths (referred to in data dictionary as CSH% etc.). Other key numerical variables analysed included the Cumulative batch number (i.e. rank of the batch by farrowing date within system based on this dataset) to measure within system effects of time (a proxy for learning), the number of days pre-farrow that sows were moved into farrowing pens and lameness and body condition scores at farrowing and weaning. Weight measurements for sows and litters were recorded at farrowing and weaning, but, apart from litter birth weight, these were missing for about 1/3rd of litters (Supplementary Materials Table S1). Categorical variables in the data (Supplementary Material – Data dictionary Table S2) included identifiers for sows and for sow by parity (the data rows for all the recorded performance data), as well as identifiers for batch, pen, and system (A, B, C, D). Other variables treated as categorical in the statistical analyses were parity, with parity 5 or more grouped together (P5+), whether the sow had a pharmacologically induced farrowing (IND), whether oxytocin (OXY) was given and whether there was savaging of one or more piglets (SAV). There were substantial missing data for these three variables, and for lameness and body condition scores (see Supplementary Materials – Data dictionary Tables S1 and S2).

Initial data checking, cleaning and exploration was carried out on the full dataset, as described in Supplementary Material - Statistical analysis - additional details. Generalised linear mixed models (GLMM) with logit link and binomially distributed residual variation were used to analyse mortality and mortality types, with response measure being the mortality count per litter (e.g. Crushed_tot: Total number of crushed piglets) and binomial total the appropriate litter size measurement (e.g. BA + F_ADJ (Total liveborn piglets after fostering adjustments to weaning– i.e. foster in and foster out– Supplementary Materials Table S1)). GLMMs with log link and Poisson distributed residual variation were used to analyse count data (e.g. BA: Total number of born alive piglets, WEANED: Number of weaned piglets). Linear mixed models (LMM) were used to analyse numerical data (e.g. weights, days pre-farrowing, parity) as well as some mortality data (appropriately transformed) when counts were low and GLMMs would not converge. Random effects included in the models were Batch, Pen, and Sow, though as less than 10% of sows had multiple litters in the dataset Sow had to be dropped from some models, particularly in GLMMs where counts were low, or other models based on fewer litters due to missing data. Candidate fixed effects fitted as categorical variables were System, Parity, SAV, OXY, IND (see Supplementary Materials– Data dictionary Table S2). Candidate fixed effects included as covariates (with linear effects on analyses scales) were numerical variables Cumulative batch number, Days_pre_farrow, weights, Lameness and body CS at Farrowing and Weaning, TEATS, and appropriate litter size measurements (see Supplementary Materials– Data dictionary Tables S1 and S2). Alternative time effects to Cumulative batch number were investigated (such as year and time in year of farrowing) but time in year effects were not consistent between systems and so, for simplicity, Cumulative batch number is reported to examine the effect of length of time that the systems had been monitored for the project. Note that the fixed effect, System, investigated here may reflect the four different types of systems, or could reflect other aspects of these particular four systems, as there is no replication in this study within each type of system. Furthermore, substantially fewer data were collected on systems B and C, and therefore, there is substantially more statistical power for comparisons between systems A and D.

GLMMs and LMMs were first investigated, and estimates produced, with single fixed effects on their own and then, for the most important response measures, models were investigated with several fixed effects included. In order to aid interpretation, estimates from the models are mean ± SE shown on back transformed scales (for example percentages, or counts,…) with explanatory covariates also back transformed. P values are based on approximate F tests when available but otherwise are based on Wald tests. See Supplementary material - Statistical analysis – additional details for more information on modelling. Data were initially collated and cleaned using Excel. Further data cleaning and processing including calculation of derived variables, exploratory and statistical analysis was conducted using code in Genstat (23rd edition, VSN International Ltd).

## Results

Over the course of the project 2920 sows produced 46,789 piglets (including 4.75% born dead) in 3212 litters across the three participating farms, with about 10% of sows recorded for two or more litters. Most of these litters were from systems A (*n* = 1646, 51.2%), and D (*n* = 1356, 42.2%), with 121 litters (3.77%) from system B (2.77%) and 89 from C. Performance data from birth to weaning were collected on the majority of sows and piglets.

### Sow characteristics

The average parity of sows was 3.03 (SD 1.52) and ranged from 1 to 7 (medians and actual counts per parity, per system are presented in Supplementary Materials Table S3). Whilst Farm 2 had lower average parity than Farms 1 and 3, this was marginally insignificant (Farm 1, system A = 3.12 ± SE 0.12; Farm 2, system B = 2.48 ± SE 0.21; Farm 3, system C = 2.96 ± SE 0.24 and system D 2.93 ± SE0.11, F_3,115_=2.31, *P* = 0.080, from LMM). Average sow body CS upon entry to the farrowing house and exit at weaning was 3.08 (SD 0.31) and 2.89 (SD 0.25) respectively. Whilst statistically there were highly significant differences between farms, numerical differences were small (respectively, estimated mean range 3.03–3.34, 2.72–2.97, F_3,90_=13.78, F_3,88_=16.44, both *P* < 0.001, from LMM). Mean body weight increased with parity (respectively, estimated mean range from parities 1 to 5+, 215.0-309.5 Kg at farrowing, 178.2-259.3 Kg at weaning, F_4,1101_=661.36, F_4,875_=414.74, both *P* < 0.001, from LMM). However, sow body weight loss (%) between farrowing and weaning was fairly similar between parities (range 16.2-18.2%, F_4,835_=3.3, *P* = 0.010, from LMM). Average lameness score was low upon entry to the farrowing house, 0.11 (SD 0.40), and at weaning, 0.17 (SD 0.50). Whilst statistically there were highly significant differences between farms, differences were numerically small (respectively, estimated mean range 0.06–0.17, 0.02–0.37, F_3,111_=6.57, F_3,99_=17.44, both *P* < 0.001, from LMM). Sows had an average of 14.2 ± SD 1.1 functional teats.

### Performance

#### Influence of system, sow condition, parity and litter size

Overall, the average percentage total mortality (TM%), live-born (LBM%) and stillborn (SB%) mortality per litter was 19.3% (SD 15.5), 15.7% (SD 14.6) and 4.3% (SD 7.5) respectively, but there were differences in KPIs between farms/systems (*P* < 0.001, Table 3). Mean percentage mortality was highest in A and lowest in D and this was mainly evident in the PRE-processing period (*P* < 0.001– Table 3). Whilst mean total numbers of piglets born (TB), and totals born alive (BA) were highest in A, and lowest in B, mean numbers weaned was lowest in A and highest in D and C. Mean percentage weaned of total born was lowest in A and highest in D (F_3,141_=58.16, *P* < 0.001, A: 79.1% B: 87.3% C: 85.1% D: 89.9% from GLMM).

**Table 3:** Average (mean ± SD) and ranges for Key Performance Indicators showing differences between farms/systems, not adjusted for any other factors. Statistical tests are from GLMMs with only system included in the fixed effects

		Total mortality (%)		Live-born mortality (%)		Stillborn mortality (%)		Total born		Born alive		Weaned		PRE live-born mortality (%)		Born alive + Fostering adjustment^*^	
		Mean (SD) (range)	n	Mean (SD) (range)	n	Mean (SD) (range)	n	Mean (SD) (range)	n	Mean (SD) (range)	n	Mean (SD) (range)	n	Mean (SD) (range)	n	Mean (SD) (range)	n
System	A	25.36	1534	20.58	1534	5.90	1627	15.42	1628	14.44	1627	10.88	1582	9.55	1613	14.10	1538
		(15.48) (0-100)		(14.89) (0-100)		(8.79) (0-94.7)		(3.66) (0–26)		(3.44) (1–24)		(1.76) (0–16)		(12.10) (0-100)		(3.03) (2–29)	
	B	15.65	118	13.21	118	2.76	121	13.39	121	12.99	121	11.50	121	8.43	121	13.28	118
		(12.41) (0-64.3)		(12.04) (0-64.3)		(4.73) (0–25.0)		(3.21) (1–20)		(3.09) (1–19)		(1.43) (7–15)		(9.96) (0–40)		(1.88) (8–20)	
	C	15.96	87	14.07	87	2.49	88	14.56	88	14.18	88	12.59	87	7.34	87	14.93	87
		(9.94) (0-53.6)		(9.59) (0-45.8)		(4.22) (0-21.1)		(3.42) (3–20)		(3.35) (3–20)		(0.87) (10–14)		(8.21) (0-33.3)		(1.88) (12–24)	
	D	12.59	1299	10.29	1299	2.69	1345	13.97	1345	13.57	1345	12.55	1292	4.82	1343	13.98	1302
		(13.09) (0-100)		(12.58) (0-100)		(5.40) (0-47.1)		(3.12) (1–24)		(3.03) (1–23)		(1.29) (0–16)		(5.40) (0–60)		(2.00) (0–26)	
F _ndf,ddf_		80.09_3,141_		58.76 _3,141_		32.35_3,182_		20.15_3,183_		9.43_3,187_		43.09_3,208_		16.30_3,136_		3.43_3,210_	
P-value		< 0.001		< 0.001		< 0.001		< 0.001		< 0.001		< 0.001		< 0.001		0.018	

ndf,ddf = numerator and denominator degrees of freedom for F statistic

^*^ Born alive + fostering adjustment = Total liveborn piglets after adjustments to weaning

Sow parity significantly affected performance with performance generally decreasing from parity 2 onwards (see detailed Results in Supplementary Materials, Table S4, Figure S2). There were some highly significant effects of sow body CS at weaning on TM% and LBM% mortality respectively, (Wald/ndf)_1_=12.56, (Wald/ndf)_1_=18.64 both *P* < 0.001, from GLMMs), with sows of higher weaning CS returning higher mortality rates. There was no significant effect on KPIs of sow lameness before farrowing (*P* = 0.381–0.853, from GLMMs) or at weaning (*P* = 0.108–0.670, from GLMMs).

Piglet mortality percentages increased markedly with litter size measures (*P* < 0.001, see detailed Results in Supplementary Materials Table S5, Figure S3).

Testing effects of system on TM%, LBM% and SB% after adjusting for parity and pertinent litter size measures resulted in decreased F-test statistics by about 10–20% (results not shown) compared to the GLMMs with system alone (Table 3). However, the adjusted tests for the system effects remain highly significant (*P* < 0.001) suggesting that there are system effects on piglet mortality that cannot be accounted for by differences in sow parity and/or litter size.

**Fig. 2:**
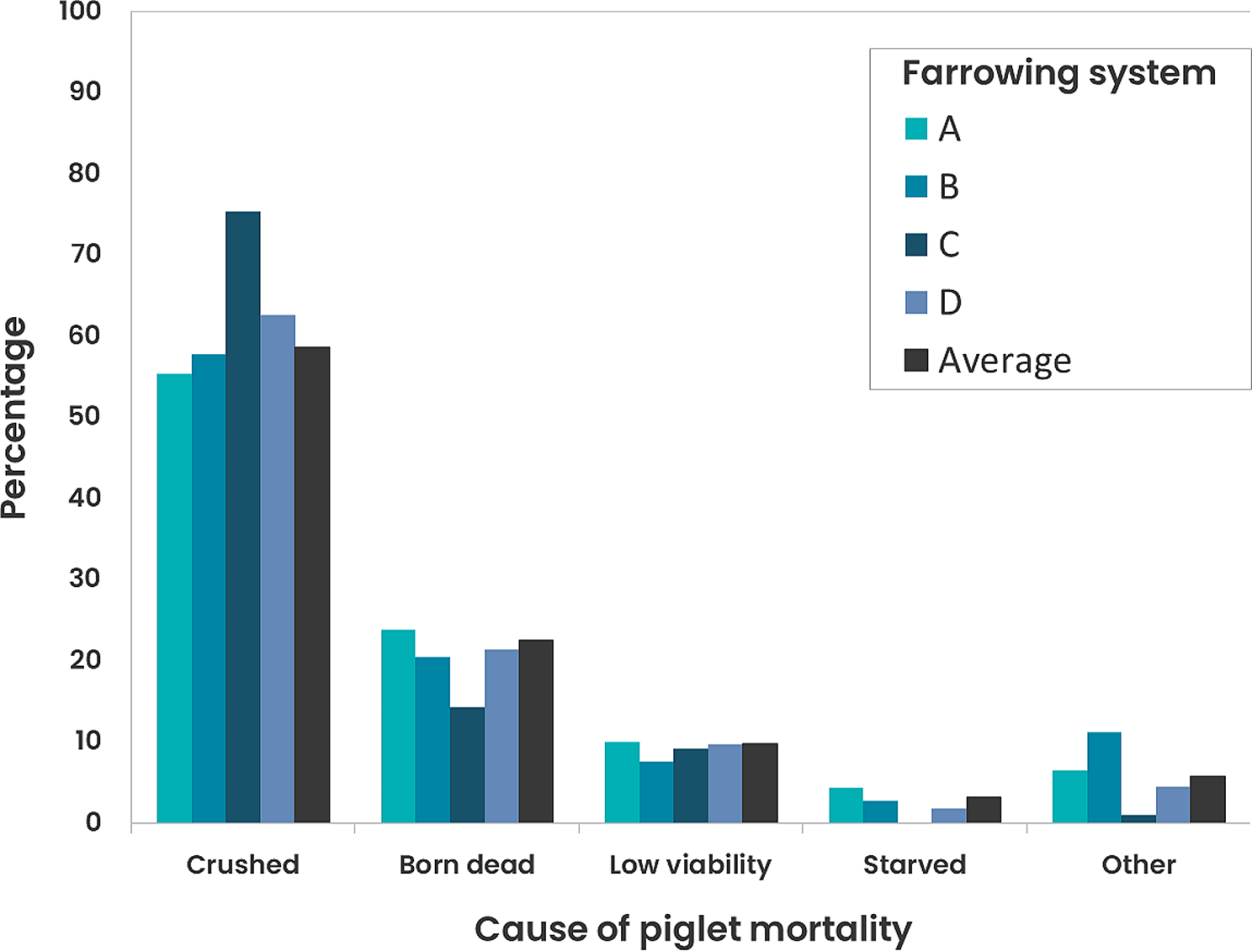
Distribution of cause of piglet mortality from birth to weaning (percentage of total litter deaths) averaged across litters in all farms (black shaded bars) and within farrowing systems (**A**, **B**, **C** and **D**)

The majority of piglet deaths on all farms were registered as crushing by the sow (58.7%±SD 37.2% of all deaths - Fig. 2). Crushing deaths as a percentage of BA adjusted for fostering (see Supplementary Material Table S1) was highest in system A (14.6% estimate from GLMM) and lowest in D (7.4% from GLMM), but as a percentage of all deaths, system C was substantially higher than all other systems, with 76.3% (from GLMM) of deaths due to crushing (Fig. 2; Table 4, Supplementary Materials Table S5, all *P* < 0.001). System also significantly influenced the percentage of all other types of mortality (all *P* < 0.001, Fig. 2; Table 4) and to a lesser extent as a percentage of all deaths (Supplementary Materials – Table S5). The percentage of piglets crushed of BA adjusted for fostering increased with parity from about 9.2% in parities 1 and 2 to 12.3% for parity 5 or more (means estimated from GLMM, *P* < 0.001, Table 4). Increases in percentage piglets crushed of BA adjusted for fostering was large (means estimated from GLMM, *P* < 0.001) from about 1.3% for five piglets to 14.5% for 17 piglets. Testing effects of system on percentage piglets crushed of BA adjusted for fostering after adjusting for parity and the pertinent litter size measure resulted in a decreased F-test statistic by 23% (results not shown) compared to the GLMMs with system alone (Table 4). However, the adjusted tests for the system effect remains highly significant (*P* < 0.001) suggesting that there are system effects on crushing that cannot be accounted for by differences in sow parity and/or litter size.

**Table 4:** Statistical tests from GLMMs for effects of system, parity and litter size on crushing and other types of mortality (as percentage of BA adjusted for fostering), not adjusted for any other factors. Bolded cells indicate dropped sow term from random effects and results for percentage Starved are from LMMs. Test statistics are (Wald/ndf)_ndf_ or F_ndf,ddf_

	Crushed (%)	Low viability (%)	Starved (%)	Other (%)	PRE-Crushed (%)	POST-Crushed (%)
System	31.91_3,139_	**6.45**_**3,188**_	10.90_3,170_	17.62_3,155_	13.45_3,140_	17.22_3,115_
	< 0.001	**< 0.001**	< 0.001	< 0.001	< 0.001	< 0.001
Parity	12.10_4_	**9.27**_**4**_	4.1_4_	5.47_4_	4.67_4_	3.62_4_
	< 0.001	**< 0.001**	0.002	< 0.001	< 0.001	0.006
TB	116.64_1_	**50.57**_**1**_	1.74_1,3036_	13.68_1_	21.92_1_	14.29_1_
	< 0.001	**< 0.001**	0.187	< 0.001	< 0.001	< 0.001
BA	107.71_1_	**47.62**_**1**_	0.32_1,3035_	12.06_1_	16.68_1_	11.89_1_
	< 0.001	**< 0.001**	0.573	< 0.001	< 0.001	< 0.001
LS_24h	17.00_1_	**366.04**_**1**_	12.40_1,2873_	2.48_1,2949_	21.42_1_	5.91_1_
	< 0.001	**< 0.001**	< 0.001	0.116	< 0.001	0.015

**Fig. 3:**
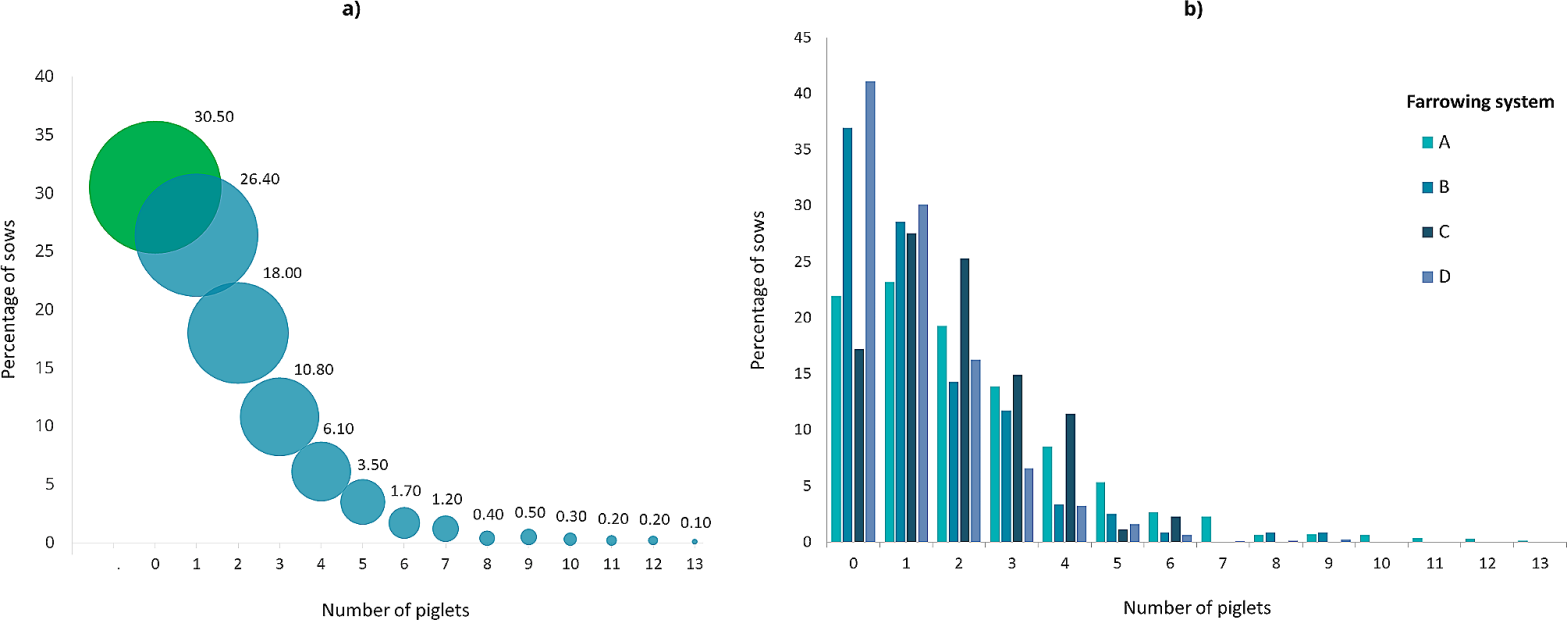
(**a**) Percentage of sow litters across all farms (*n* = 3074) that crushed no piglets (0, green circle) or crushed between 1–13 piglets (blue circles). The area of the circles represents the number of sows. (**b**) Percentage of sow litters in each farrowing system (**A**, **B**, **C**, **D**) that crushed no piglets (0) or crushed between 1–13 piglets

When looking at the number of crushed piglets per litter, this ranged from 0 to 13, however the majority of sows (57%, *n* = 1751) crushed 0 or just one piglet (Fig. 3a), with the highest numbers crushed per litter (13) coming from two litters from Farm 1 (system A). There were significant differences between systems in the number of crushed piglets per litter (*P* < 0.001, F_3,142_=34.68 from GLMM; Fig. 3b) with the most piglets crushed in A and the least in D (means estimated from GLMM: A 2.1, B 1.3, C 1.8 and D 1.0).

#### Influence of length of time system operational on farm

**Fig. 4:**
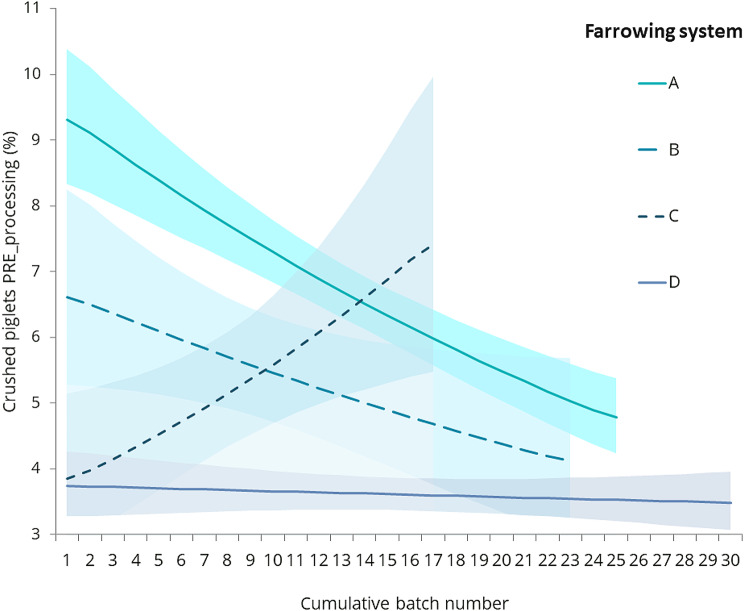
Effect of interaction (*P* = 0.037) between system and time (Cumulative batch number) on the percentage of piglets crushed PRE-processing. Lines show means from GLMMs and shaded areas show the means ± upper and lower standard errors (all back transformed)

There was a marginally statistically significant effect of Cumulative batch number within system on PRE-processing LBM% (F_1,73_=5.29, *P* = 0.024), but not POST-processing LBM% (F_1,74_=0.01, *P* = 0.940). There was a marginally statistically significant effect of the Cumulative batch number within system on the percentage of piglets crushed in total and PRE-processing, but not POST-processing (Crushed %, F_1,77_=6.0, *P* = 0.017, PRE-Crushed %, F_1,74_=7.48, *P* = 0.008, POST-Crushed %, F_1,71_=0.30, *P* = 0.585, from GLMMs with Cumulative batch number only) with less crushing over time by about 4% (from GLMM) over the four systems. There was a marginally statistically significant interaction between System and Cumulative batch number for the percentage of piglets crushed in the PRE-processing period (F_3,165_=2.90, *P* = 0.037; Fig. 4), which suggested that crushing was steady and low for System D (at just under 4%), and initially high but decreasing for System A (from about 9–5%) and B, but increasing for System C. However, the SEs for systems B and C are very large (Fig. 4) and so evidence for the observed trends for those two systems is weak.

#### Influence of farrowing management

**Fig. 5:**
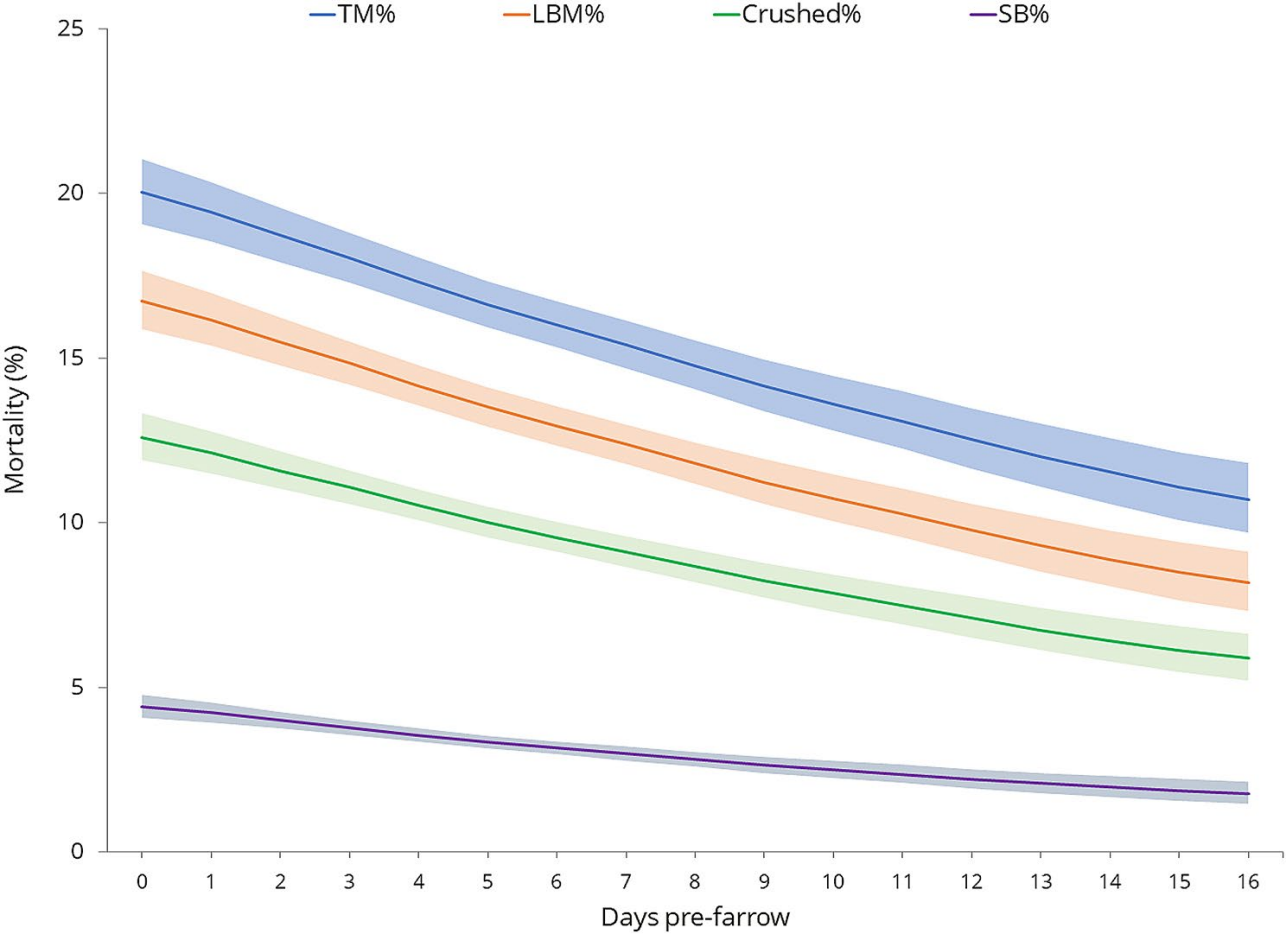
Effect of length of time (days) between moving in to farrowing accommodation and farrowing on piglet mortality (*P* < 0.001). Lines show means from GLMMs and the shaded areas the means ± upper and lower standard errors (all back transformed)

There was a highly significant effect on piglet mortality of length of time (days) between moving sows into farrowing accommodation and sows giving birth (TM%: (Wald/ndf)_1_=30.01; LBM%: (Wald/ndf)_1_=30.49; SB%: F_1,1130_=15.03; Crushed %: F_1,2809_=27.68; PRE-Crushed %: F_1,2209_= 24.71; POST-Crushed %: F_1,2781_=7.21, all *P* < 0.001, from GLMMs – Fig. 5). The less time between move in and farrowing, the higher the levels of mortality, by about 3% total mortality every five days.

There were some system differences in days pre-farrowing (F_3,92_=21.99; *P* < 0.001, from LMM). System A and C averaged the shortest length of time between moving in and farrowing at 3.04 ± SD 1.65 and 3.69 ± SD 2.67 days respectively and system B and D averaged the longest time at 5.26 ± SD 2.03 and 5.53 ± SD 2.52 respectively. Testing effects of system on TM%, LBM%, SB%, Crushed % (Total, PRE and POST) after adjusting for parity, pertinent litter size measures, and days pre-farrowing resulted in decreased F-test statistics by about 25–50% (results not shown) compared to the GLMMs with system alone (Tables 3 and 4). However, the adjusted tests for the system effects remain highly significant (*P* < 0.001) suggesting that there are system effects on piglet mortality that cannot be accounted for by differences in sow parity, litter size and/or days pre-farrowing. Effects of pertinent litter size measures and parity are fairly similar in the GLMMs with all four fixed effects and the GLMMs with these two measures alone. Effects of days pre-farrowing are highly significant when tested after parity and litter size (P = < 0.001–0.003), but more marginal when tested last after system (SB% *P* = 0.536, TM% *P* = 0.027, LBM% *P* = 0.018, Crushed % *P* = 0.041, POST-Crushed % *P* = 0.258), apart from for PRE-Crushed % (*P* < 0.001), for which the effect of days pre-farrowing is similar in GLMMs with all four terms and with days pre-farrowing alone. This suggests that whilst differences in days pre-farrowing are to some extent a between system effect, there is also evidence that days pre-farrowing affects mortality within systems, particularly on percentage of crushed piglets pre-processing.

There were statistically significant interactions between system and days pre-farrowing on mortality (TM%: (Wald/ndf)_3_=6.61, *P* < 0.001; LBM%: (Wald/ndf)_3_=3.91, *P* = 0.008; SB%: (Wald/ndf)_3_=3.02, *P* = 0.029; Crushed %, (Wald/ndf)_1_=4.04, *P* = 0.007 from GLMMs). Mortality decreased markedly with days pre-farrowing for system A, but remained fairly steady for system D (SEs for B and C are very large). Note that this interaction was not significant for PRE-Crushed % ((Wald/ndf)_1_=0.90, *P* = 0.441, from GLMM) suggesting that percentage of crushed piglets pre-processing decreased with days pre-farrowing similarly in all four systems.

Savaging, induction and use of oxytocin was only measured for about 60% of the litters and rarely measured for farms C and D. Percentage of litters for which savaging was observed was 1.2%, whilst use of oxytocin was 8.9% and induction 37.6% of litters both mainly in Farm A. Savaging, as expected, affected mortality (TM%: F_1,1418_=5.67, *P* = 0.017; LBM%: F_1,1322_=9.02 *P* = 0.003), predominantly in the PRE-processing period (PRE-TM%: F_1,1498_=5.59, *P* = 0.018; PRE-LBM%: F_1,1192_=11.97, *P* = 0.001, all from GLMMs).

## Discussion

Our results demonstrate that most domestic sows can perform well in these free farrowing systems and that results can improve with time, although there are system effects suggesting that certain systems are more challenging for successful free farrowing. It is also important to note that the system effects observed here may reflect the four different designs of farrowing system, or could reflect other management aspects of these particular four systems, as there is no replication in this study of systems across farms. Furthermore, substantially fewer data were collected on systems B and C, and therefore, statistically significant system differences observed were most frequently as a result of differences between systems A and D.

The study recorded sows on commercial units with three newly installed systems and one established system. Total mortality of all piglets in the study was 19.3%, which included 4.3% stillbirths. Comparative commercial data from indoor free farrowing herds are sparse but can be found from countries that have banned farrowing crate use. In Norway, figures from a 2018 study on 14 commercial farms reported total mortality at 23.4%, including 6.3% stillbirths, with average total born litter size calculated from their figures as 15.14 piglets [28]. In a Swiss study, Weber et al. [4] reported live-born mortality between 11.5 and 13.5% from 255 farms where average born alive litter size was 12.8 piglets. A Swedish study in 2019 [29] followed 318 litters and reported 20.9% live-born mortality from an average born alive litter size of 14.3 piglets. Live-born mortality for the four systems in our study ranged from 10.3 to 20.6% from born alive litter sizes ranging from 13.0 to 14.4 piglets. Thus, our results are in line with the range of figures reported by these studies.

Improvement over time was evident (averaged over the four systems), supporting the notion that a transition period is to be expected when adopting a new free farrowing system, as documented in other studies, particularly in commercial trials [3, 30]. There was, however, an interesting interactive effect of system and cumulative batch (i.e. time) on the amount of crushing mortality observed in the first 24 h post-partum, with crushing being steady and low for the established system (D), decreasing markedly for one of the newly installed free farrowing systems (A), decreasing somewhat for B, but increasing for system C. However, it should be noted that the standard errors for systems B and C were very large and so evidence for the observed trends for those two systems is relatively weak, possibly due to data being collected on substantially fewer litters.

Farrowing system was shown to influence the levels of different types of mortality. Whilst crushing mortality was the predominant cause of death on all farms in this study (agreeing with reports from other studies [31–33]), the levels of crushing differed. Despite Farm 1, system A recording the highest mortality levels in the study, they did not record the highest value for crushing as a percentage of total mortality. This was registered on Farm 3, predominantly by system C. This was the newly installed temporary crate system (the ‘360’), operated in its open position for the trial. It was the smallest system (occupying the same footprint as a conventional farrowing crate) and such limited space influences how easily sows can avoid making contact with their litter when changing posture. This is something noted by other authors using temporary crating systems in their open position for farrowing [34]. In addition, the creep area in system C was not clearly defined or enclosed (which it was for the other three systems). Given the choice sows would delineate space into areas for resting/farrowing/nursing, eating and elimination [8, 22, 35] and providing covered creeps has been shown in some studies to reduce crushing mortality [36]. This lack of defined functional space for both sows and piglets could have increased the risk of piglets becoming trapped during sow posture changes, which is something noted by other authors who associated small nest spaces and narrow widths with higher piglet mortality in their studies [7, 34, 37]. The dimensions of the nest space are likely to influence the quality of mother-young interactions, particularly how well sows are able to turn and group or cluster their piglets during the pre-lying period, which is a behaviour associated with carefulness [38, 39]. As well as the lack of avoidance space, it is possible the sows in system C were more restless during farrowing, as a result of the lack of appropriate substrate (sows in C only received some shredded paper) as well as limited space to perform satisfactory nest-building behaviour. Thwarted nest-building behaviour can increase stress (recorded as elevated plasma cortisol and ACTH concentrations in crated as opposed to free farrowing sows [40, 41]) and reduce circulating levels of hormones, such as oxytocin, known to modulate maternal nurturing behaviour [9, 42]. Behavioural observations of sows in these systems would offer more insight to support these discussions and any comparisons should be treated with caution as there were many differences between farms and systems that could affect performance, including specific system designs and farrowing room factors, genetics, stockperson skill level and experience of both staff and sows. This latter point is highly influential on how well alternative systems perform [3, 43, 44] and is particularly critical as more farms transition from farrowing crates to less confined farrowing systems and are operating multiple systems on farm that both staff and sows alternate between. There are risks with this strategy as noted by King et al. [43, 44] in other work conducted within this project. They demonstrated the effect of previous farrowing system on current performance, noting that sows farrowing in the same system for repeated parities showed lower piglet mortality than those swapping between systems, and emphasised the importance of consistency in order to achieve sustained good performance. When returned to the same farrowing system as that experienced during the previous farrowing, sows show adapted farrowing behaviours including reduced restlessness [45]. Stockpeople are also likely to adapt and optimise their husbandry practices to enhance performance. In the current study, one practice (of those measured) was highlighted as detrimental to piglet survival; late entrance into the farrowing accommodation. This agrees with previous research [46, 47] and it is likely that late entrance into farrowing accommodation is disruptive to maternal behaviours that can enhance piglet survival, namely nest-building. The level of disturbance farrowing sows might experience is also likely to impact maternal behaviour. The natural behaviour of an expectant sow is to seek a nest-site that is isolated from the main herd and potential predators. Such a site minimises risk of disturbance, thus minimising sow posture changes risking piglet crushing during and after farrowing, allowing a sustained period of contact between mother and young, facilitating successful suckling and the formation of essential bonds before reintegration with the main herd [8, 10]. Such an ideal nest-site is difficult to replicate under indoor commercial conditions, even in well designed free farrowing accommodation, as disturbance from neighbouring sows and stockperson activity are almost inevitable. The level of disturbance is influenced by pen design and the number of pens per farrowing room; too many pens per room can be disruptive and hyper-responsive sows maybe particularly sensitive to their neighbours [18]. Disturbance during parturition is known to negatively influence oxytocin levels [48], could influence the length of the important non-responsive phase after the birth of the last piglet [11] which facilitates safe udder access for piglets and may influence subsequent restlessness of the sow and risk of piglet crushing. In this study, there was high potential for disturbance particularly on Farm 1, system A where there were 72 pens per room and on Farm 3 in system C where the 360s were in close proximity to each other, had low pen sides and there were 20 per room. Farm 2, system B only had 4 pens per room and Farm 3, system D had the potential for the least disturbance with sows essentially singly housed in high-sided kennels with runs. This highlights the importance of design details, not only for the individual pen but also for the overall farrowing room [18].

Regardless of farrowing system, all key performance indicators were heavily influenced by litter size and age of the sow. Large litters are well-known to be a major risk factor for early piglet mortality [49–52]. Increasing litter size is often accompanied by a reduction in average birth weight, including a higher proportion of piglets showing intrauterine growth retardation (IUGR) [53] and increased numbers of stillborn piglets. Lower birth weight is associated with greater mortality and IUGR piglets in particular can require targeted interventions by staff if they are to survive [54]. Regardless of the robustness of the piglet, when sows consistently produce more piglets than functional teat space, interventions by stockpeople are paramount to ensure early colostrum intake and maximise piglet survival [54]. This is challenging in conventional systems but could be more so when the sow is loose and stockpeople have little experience with free farrowing management. This was the case with Farms 1 and 2. However, Farm 2 only trialled 8 pens (system B), whereas Farm 1 was operating 72 pens (system A) in one barn, had the highest numbers born alive (14.44, range 1–24) and returned the highest live-born piglet mortality (20.6%). Average teat number on this farm was 14.1 ± SD 0.9.

The parity effect we observed in this study (older sows having higher mortality) may partly be explained by the increase in litter size observed with increasing parity. Farm 1 had higher average parity compared to the other farms and had a higher level of stillbirths. Older sows tend to give birth to more piglets and more stillborn piglets [32, 49, 55, 56]. Older sows may also have fewer functional teats, which are less accessible, especially to low viability piglets, and may have a reduced milk yield [57]. Compared to young sows, older sows have a larger mass and body weight, often accompanied with reduced mobility, which are all heightened risk factors for damaging crushing of piglets or a failure to respond to the calls of a trapped piglet [58]. Sow weight could only be accurately recorded on a subset of animals, but body condition score was recorded on the majority and the observation that sows of higher weaned body condition score returned higher piglet mortality rates may support the notion of heavier sows being less mobile, with greater potential to damage their piglets. Alternatively, this result may reflect that higher mortality means fewer piglets nursed and so less body tissue mobilisation occurred for those sows. It is likely a combination of sow, piglet and environmental factors influencing these results that are difficult to disentangle. Observing the behaviour of sows of different body condition scores to determine their mobility around their piglets would more fully inform this discussion, as would observations of piglet behaviour.

## Conclusion

These results from commercial farms demonstrate that even sows that have not been specifically selected for free farrowing are able, in many cases, to perform well in zero-confinement systems, but that a period of adaptation is to be expected for overall farm performance. In addition these results suggest it would be more challenging to operate a system designed for temporary crating, with limited space, as a zero-confinement system since the risks of crushing are particularly high. Therefore, systems that afford more space, and are designed with the mindset that the sow will be loose throughout, could be less challenging to operate in the long-term. However, some caution is required when drawing conclusions about system designs, as there was no replication of the types of systems across the different farms.

Optimising management protocols can improve piglet survival. In particular, giving sows enough time in their farrowing accommodation prior to their due date has been highlighted as a key management procedure to reduce piglet mortality.

Crushing remains the main cause of piglet death, but many sows do not crush any piglets. Providing more information on the behaviour and physiology of these successful free farrowing sows and their offspring should allow for the optimisation of breeding programmes to select for maternal traits for sows which will perform well in free farrowing systems. However, system design and management of large litter sizes should also be improved, given the high influence these factors have on performance. Genetic selection for prolificacy has been driven by economic considerations, but poses particular challenges in zero-confinement systems.

### Electronic supplementary material

Below is the link to the electronic supplementary material.

Supplementary Material 1

## Data Availability

The datasets used for producing the results presented in this study are available from the corresponding author upon reasonable request.

## Footnotes

1 https://ahdb.org.uk/knowledge-library/body-condition-scoring-sows.
