# Supplementary material for: Factors contributing to high performance of sows in free farrowing systems
Source: Porcine Health Manag. 2024 May 2;10:16. doi: 10.1186/s40813-024-00366-w (PMC11064381; doi:10.1186/s40813-024-00366-w)
Supplement: Supplementary file 1 — Supplementary Material 1 [file 40813_2024_366_MOESM1_ESM.docx]

**Supplementary Materials**

**Animal housing and management – additional details**

Figure S1 shows pictures of the four different systems (A, B, C, D) on the three different farms used in the trial. Figure 1 in the main manuscript gives detailed measurements.


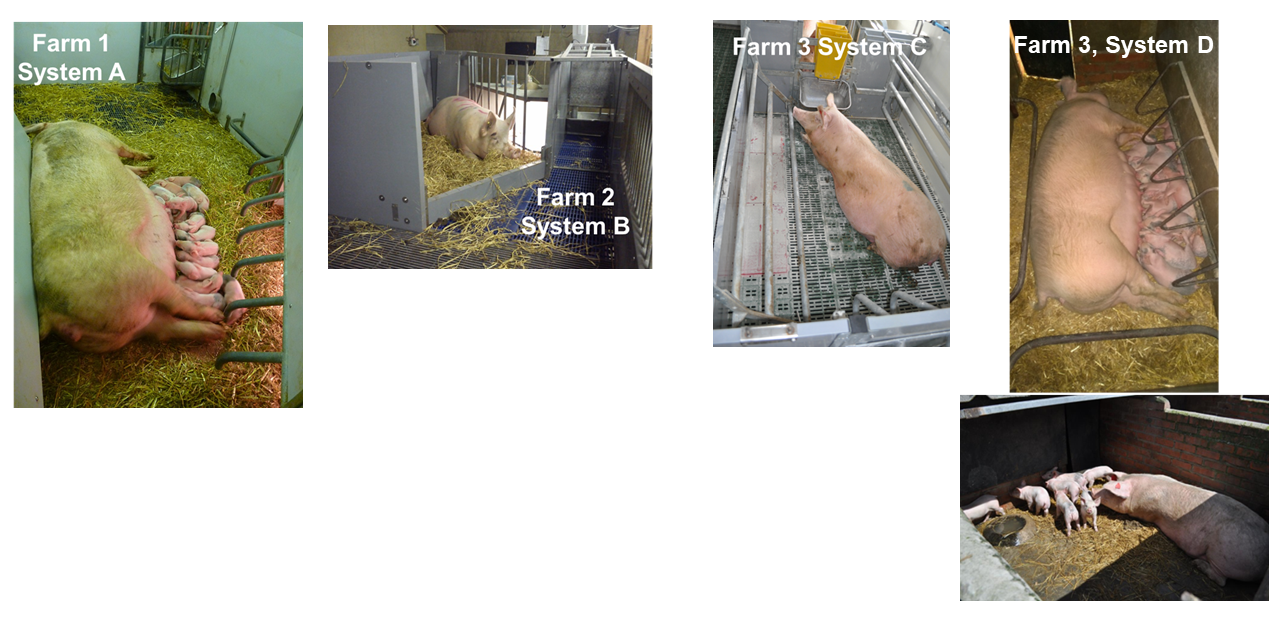


**Figure S1.** Pictures of the free farrowing systems studied on the three commercial farms. Farm 1 operated system A) (a Danish Free Farrower type design), Farm 2 operated system B) (PigSAFE design) and Farm 3 operated both systems C) (a ‘360^®^’ temporary crate in its open position for the trial) and D) (a ‘Solari’ system with an indoor kennel area with a nest site and creep for the piglets (top picture) and an outdoor run (bottom picture)). All pictures provided by authors.

**Statistical analysis – additional details**

Initial data cleaning and exploration generated summary statistics and histograms to examine distributions of numerical variables, and code to check internal consistency and derived data. Appropriate graphs and tests were used to screen for bivariate relationships between numerical variables (using correlations), between numerical and categorical variables (using ANOVA), and between categorical variables (using a Chi-squared permutation test). From this preliminary analysis, repeated once data had been cleaned, some of the descriptive statistics (e.g. mean±SD) are given in the results, as well as some Spearman’s correlation coefficients (r_s_). These preliminary analyses were used as a guide to subsequent modelling, for example to determine the extent of missing data, which random effects were likely estimable, and whether binomial or count data were too sparse for inclusion of some categorical fixed effects, as well as candidate explanatory variables for each response variable. GLMMs and LMMs were first investigated, and estimates produced, with single fixed effects on their own. This is crucial because as this is observational data, there is confounding between candidate fixed effects, and models with multiple confounded fixed effects can produce unstable estimates. Then, for the most important response measures, models were investigated with several fixed effects included, and sequential tests investigated in these models as well as checking the resulting estimates against those from models including single fixed effects. The sequential tests can be used to address questions of interest such as whether system effects are still significant after adjusting for expected differences between systems, such as in litter size, parity, and days pre-farrowing. Some response measures in LMMs were transformed (e.g. log for counts, angular for percentages) to improve normality and homogeneity of residual variability. Explanatory covariates were also similarly transformed, for example, logs of counts, and then standardised to improve model stability (by subtracting the mean and dividing by the SD). In order to aid interpretation, estimates from the LMMs and GLMMs are mean±SE shown on back transformed scales (for example percentages, or counts, …) with explanatory covariates also back transformed. Note that relationships with covariates may not appear linear once back transformed. P values are based on approximate F tests when available but otherwise are based on Wald tests. F statistics are given in the results for F tests along with numerator and denominator degrees of freedom (ndf and ddf), and, when Wald tests are used, Wald/ndf along with numerator degrees of freedom are given as this is comparable with the F statistic. Broadly, the larger the value the greater the evidence for an effect and so it is useful to compare magnitudes of these statistics especially when there are several highly significant effects. Note that, as this dataset is observational, statistically significant effects do not necessarily imply causality.

**Data dictionaries**

**Table S1.** Data dictionary showing the numeric variables in the dataset, including definitions, equations for derived variables, number of observations (nobvs), number of missing values (nmvxxx), number of non-missing values (nvalxxx) and percentage of missing values.

| **Variable name** | **Definition** | **Equation for derived variables** | **nobs** | **nmvxxx** | **nvalxxx** | **% missing values** |
| --- | --- | --- | --- | --- | --- | --- |
| Cumulative_batch | Cumulative batch number (i.e. ranked by farrow date) within Farm |  | 3212 | 0 | 3212 | 0.0 |
| Days_pre_farrow | Number of days pre-farrowing that sows were moved in to farrowing accommodation |  | 3128 | 84 | 3212 | 2.6 |
| Farrow_date | Farrowing date |  | 3191 | 21 | 3212 | 0.7 |
| FW | Sow weight pre-farrowing (kg) |  | 1270 | 1942 | 3212 | 60.5 |
| FCS | Sow body condition score pre-farrowing |  | 2794 | 418 | 3212 | 13.0 |
| F_Lame | Sow lameness score pre-farrowing |  | 2508 | 704 | 3212 | 21.9 |
| TB | Total number of piglets born in litter |  | 3182 | 30 | 3212 | 0.9 |
| BA | Total number of born alive piglets |  | 3181 | 31 | 3212 | 1.0 |
| LBW | Litter birth weight (kg) |  | 2671 | 541 | 3212 | 16.8 |
| LBWperpiglet | Average piglet birth weight (kg) | LBW/BA | 2667 | 545 | 3212 | 17.0 |
| Wean_date | Wean date |  | 3098 | 114 | 3212 | 3.5 |
| Wean_age | Piglet weaning age (days) |  | 3094 | 118 | 3212 | 3.7 |
| WEANED | Number of weaned piglets |  | 3082 | 130 | 3212 | 4.0 |
| Total_Weaned % | Percentage of liveborn piglets weaned | 100*WEANED/(BA+F_ADJ) | 3005 | 207 | 3212 | 6.9 |
| WLW | Litter weaning weight (kg) |  | 1131 | 2081 | 3212 | 64.8 |
| WLWperpiglet | Average weaned piglet weight (kg) | WLW/WEANED | 1131 | 2081 | 3212 | 64.8 |
| WW | Sow weight at weaning (kg) |  | 918 | 2294 | 3212 | 71.4 |
| WCS | Sow body condition score at weaning |  | 2367 | 845 | 3212 | 26.3 |
| WLAME | Sow lameness score at weaning |  | 2321 | 891 | 3212 | 27.7 |
| Wgt_loss | Sow weight change (kg) | FW-WW | 899 | 2313 | 3212 | 72.0 |
| Wgt_loss% | Percentage sow weight change | 100*(FW-WW)/FW | 899 | 2313 | 3212 | 72.0 |
| BD | Total number of born dead piglets |  | 3181 | 31 | 3212 | 1.0 |
| M | Total number born mummified |  | 3181 | 31 | 3212 | 1.0 |
| TEATS | Number of sow functional teats |  | 2608 | 604 | 3212 | 18.8 |
| LS_24h | Litter size after processing |  | 3156 | 56 | 3212 | 1.7 |
| F_OFF | Number of piglets fostered off litter |  | 3156 | 56 | 3212 | 1.7 |
| F_ON | Number of piglets fostered on to litter |  | 3155 | 57 | 3212 | 1.8 |
| F_ADJ | Fostering adjustment | F_ON-F_OFF | 3155 | 57 | 3212 | 1.8 |
| LS_24h_F_ADJ | Litter size POST-processing to weaning | LS_24h+F_ADJ | 3048 | 164 | 3212 | 5.1 |
| TB_F_ADJ | Total number of piglets after adjustments to weaning | TB+F_ADJ | 3045 | 167 | 3212 | 5.2 |
| BA_F_ADJ | Total liveborn piglets after adjustments to weaning | BA+F_ADJ | 3045 | 167 | 3212 | 5.2 |
| BD_DEATHS_1 | Number of deaths PRE-processing | BD+DEATHS_1 | 3164 | 48 | 3212 | 1.5 |
| DEATHS_1 | Number of live-born deaths PRE-processing |  | 3166 | 46 | 3212 | 1.4 |
| CSH_1 | Number of piglets crushed PRE-processing |  | 3168 | 44 | 3212 | 1.4 |
| LV_1 | Number of piglets dead from low viability PRE-processing |  | 3166 | 46 | 3212 | 1.4 |
| STV_1 | Number of piglets starved PRE-processing |  | 3166 | 46 | 3212 | 1.4 |
| OTH_1 | Number of piglets dead from other causes PRE-processing |  | 3166 | 46 | 3212 | 1.4 |
| DEATHS_2 | Number of live-born deaths POST-processing to weaning |  | 3077 | 135 | 3212 | 4.2 |
| CSH_2 | Number of piglets crushed POST-processing to weaning |  | 3079 | 133 | 3212 | 4.1 |
| LV_2 | Number of piglets dead from low viability POST-processing to weaning |  | 3078 | 134 | 3212 | 4.2 |
| STV_2 | Number of piglets starved POST-processing to weaning |  | 3078 | 134 | 3212 | 4.2 |
| OTH_2 | Number of piglets dead from other causes POST-processing to weaning |  | 3077 | 135 | 3212 | 4.2 |
| All_deaths | Total number of all deaths | BD+DEATHS_1+DEATHS_2 | 3070 | 142 | 3212 | 4.4 |
| All_LB_deaths | Total number of all live born deaths | DEATHS_1+DEATHS_2 | 3070 | 142 | 3212 | 4.4 |
| TM% | Percentage Total Mortality | 100*(BD+DEATHS_1+DEATHS_2)/(TB+F_ADJ) | 3038 | 174 | 3212 | 5.4 |
| PRE_TM% | Percentage PRE-processing Total Mortality | 100*(BD+DEATHS_1)/TB | 3164 | 48 | 3212 | 1.5 |
| LBM% | Percentage Live Born Mortality | 100*(DEATHS_1+DEATHS_2)/(BA+F_ADJ) | 3038 | 174 | 3212 | 5.4 |
| PRE_LBM% | Percentage PRE-processing Live Born Mortality | 100*(DEATHS_1)/BA | 3164 | 48 | 3212 | 1.5 |
| POST_LBM% | Percentage POST-processing Live Born Mortality | 100*DEATHS_2/(LS_24h+F_ADJ) | 3040 | 172 | 3212 | 5.4 |
| SB% | Percentage Stillborn mortality | 100*BD/TB | 3181 | 31 | 3212 | 1.0 |
| Crushed_tot | Total number of crushed piglets | CSH1+CSH2 | 3074 | 138 | 3212 | 4.3 |
| LV_tot | Total number of low viability piglets | LV_1+LV_2 | 3071 | 141 | 3212 | 4.4 |
| STV_tot | Total number of starved piglets | STV_1+STV_2 | 3071 | 141 | 3212 | 4.4 |
| OTH_tot | Total number of piglet deaths from other causes | OTH_1+OTH_2 | 3070 | 142 | 3212 | 4.4 |
| Tot_Crushed% | Percentage of live-born and fostered piglets crushed in total | 100*(CSH_1+CSH_2)/(BA+F_ADJ) | 3040 | 172 | 3212 | 5.4 |
| Tot_LV% | Percentage of live-born and fostered piglets dying of low viability in total | 100*(LV_1+LV_2)/(BA+F_ADJ) | 3039 | 173 | 3212 | 5.4 |
| Tot_STV% | Percentage of live-born and fostered piglets dying of starvation in total | 100*(STV_1+STV_2)/(BA+F_ADJ) | 3039 | 173 | 3212 | 5.4 |
| Tot_OTH% | Percentage of live-born and fostered piglets dying from other causes in total | 100*(OTH_1+OTH_2)/(BA+F_ADJ) | 3038 | 174 | 3212 | 5.4 |
| PRETot_Crushed% | Percentage of live-born piglets crushed PRE-processing | 100*CSH_1/BA | 3166 | 46 | 3212 | 1.4 |
| PRETot_LV% | Percentage of live-born piglets dying of low viability PRE-processing | 100*LV_1/BA | 3164 | 48 | 3212 | 1.5 |
| PRETot_STV% | Percentage of live-born piglets dying of starvation PRE-processing | 100*STV_1/BA | 3164 | 48 | 3212 | 1.5 |
| PRETot_OTH% | Percentage of live-born piglets dying from other causes PRE-processing | 100*OTH_1/BA | 3164 | 48 | 3212 | 1.5 |
| POSTTot_Crushed% | Percentage of live-born and fostered piglets crushed POST-processing | 100*CSH_2/(LS_24h+F_ADJ) | 3042 | 170 | 3212 | 5.3 |
| POSTTot_LV% | Percentage of live-born and fostered piglets dying of low viability POST-processing | 100*LV_2/(LS_24h+F_ADJ) | 3041 | 171 | 3212 | 5.3 |
| POSTTot_STV% | Percentage of live-born piglets and fostered dying of starvation POST-processing | 100*STV_2/(LS_24h+F_ADJ) | 3041 | 171 | 3212 | 5.3 |
| POSTTot_OTH% | Percentage of live-born and fostered piglets dying from other causes POST-processing | 100*OTH_2/(LS_24h+F_ADJ) | 3040 | 172 | 3212 | 5.4 |
| BD% | Percentage mortality due to stillbirth | 100*BD/(DEATHS_1+DEATHS_2+BD) | 2580 | 632 | 3212 | 19.7 |
| Crushed% | Percentage mortality due to crushing | 100*(CSH_1+CSH_2)/(DEATHS_1+DEATHS_2+BD) | 2580 | 632 | 3212 | 19.7 |
| LV% | Percentage mortality due to low viability | 100*(LV_1+LV_2)/(DEATHS_1+DEATHS_2+BD) | 2580 | 632 | 3212 | 19.7 |
| STV% | Percentage mortality due to starvation | 100*(STV_1+STV_2)/(DEATHS_1+DEATHS_2+BD) | 2580 | 632 | 3212 | 19.7 |
| OTH% | Percentage mortality due to other causes | 100*(OTH_1+OTH_2)/(DEATHS_1+DEATHS_2+BD) | 2580 | 632 | 3212 | 19.7 |
| PRE-Crushed% | Percentage PRE-processing liveborn mortality due to crushing | 100*CSH_1/DEATHS_1 | 1635 | 1577 | 3212 | 49.1 |
| PRE-LV% | Percentage PRE-processing liveborn mortality due to low viability | 100*LV_1/DEATHS_1 | 1635 | 1577 | 3212 | 49.1 |
| PRE-STV% | Percentage PRE-processing liveborn mortality due to starvation | 100*STV_1/DEATHS_1 | 1635 | 1577 | 3212 | 49.1 |
| PRE-OTH% | Percentage PRE-processing liveborn mortality due to other causes | 100*OTH_1/DEATHS_1 | 1635 | 1577 | 3212 | 49.1 |
| POST-Crushed% | Percentage POST-processing liveborn mortality due to crushing | 100*CSH_2/DEATHS_2 | 1756 | 1456 | 3212 | 45.3 |
| POST-LV% | Percentage POST-processing liveborn mortality due to low viability | 100*LV_2/DEATHS_2 | 1756 | 1456 | 3212 | 45.3 |
| POST-STV% | Percentage POST-processing liveborn mortality due to starvation | 100*STV_2/DEATHS_2 | 1756 | 1456 | 3212 | 45.3 |
| POST-OTH% | Percentage POST-processing liveborn mortality due to other causes | 100*OTH_2/DEATHS_2 | 1756 | 1456 | 3212 | 45.3 |

**Table S2.** Data dictionary showing the categorical variables in the dataset including the number of levels, the levels, number of observations (nobvs), number of missing values (nmvxxx), number of non-missing values (nvalxxx) and percentage of missing values.

| **Variable name** | **Definition** | **Number of levels** | **Levels** | **nobs** | **nmvxxx** | **nvalxxx** | **% missing values** |
| --- | --- | --- | --- | --- | --- | --- | --- |
| FarmSowid_Parity | Sow Identifier by Parity (data row) | 3211 |  | 3212 | 0 | 3212 | 0.0 |
| FarmSowid | Sow Identifier | 2920 |  | 3212 | 0 | 3212 | 0.0 |
| Parity | Parity | 5 | (1,2,3,4,>=5) | 3212 | 56 | 3156 | 1.7 |
| Farm | Farm | 4 | (A,B,C,D) | 3212 | 0 | 3212 | 0.0 |
| Batch | Batch | 95 |  | 3212 | 0 | 3212 | 0.0 |
| Cumulative_batch | Cumulative batch number within Farm | 31 |  | 3212 | 0 | 3212 | 0.0 |
| Pen | Farrowing pen | 161 |  | 3212 | 0 | 3212 | 0.0 |
| SAV | Sow savaged piglets | 2 | (No,Yes) | 3212 | 1323 | 1889 | 41.2 |
| OXY | Oxytocin was given | 2 | (No,Yes) | 3212 | 1330 | 1882 | 41.4 |
| IND | Farrowing was induced | 2 | (No,Yes) | 3212 | 1330 | 1882 | 41.4 |

**Results – additional details**

**Sow characteristics:**

Median parity across all farms was 3. Per system median parity was: A = 3, B = 2, C = 3, D = 3. Table S3 shows the total counts of sows by parity, the percentage of the total population (‘All’) and by system.

**Table S3.** Number (n) and percentage of sows per parity and by system.

|  |  |  |  | **Farm/System** | | | | | | | |
| --- | --- | --- | --- | --- | --- | --- | --- | --- | --- | --- | --- |
|  |  | **All** | | **A** | | **B** | | **C** | | **D** | |
|  |  | **n** | **%** | **n** | **%** | **n** | **%** | **n** | **%** | **n** | **%** |
| **Parity** | **1** | 713 | 22.59 | 314 | 19.10 | 51 | 42.15 | 14 | 15.73 | 334 | 25.65 |
|  | **2** | 605 | 19.17 | 318 | 19.34 | 15 | 12.40 | 30 | 33.71 | 242 | 18.59 |
|  | **3** | 545 | 17.27 | 296 | 18.00 | 14 | 11.57 | 13 | 14.61 | 222 | 17.05 |
|  | **4** | 495 | 15.68 | 299 | 18.19 | 16 | 13.22 | 10 | 11.24 | 170 | 13.06 |
|  | **>=5** | 798 | 25.29 | 417 | 25.36 | 25 | 20.66 | 22 | 24.72 | 334 | 25.65 |

**Parity and litter size effects:**

Mortality increased with parity (Table S4, Figure S2). SB% increased with parity (P<0.001), whereas TM% was lower for parity 2 than 1, and then increased from parity 2 onwards (P<0.001) with a similar, albeit more marginal (P=0.024), pattern for LBM%. Both TB and BA, were higher from parity 3 onwards, by one piglet on average (P<0.001). The number of weaned piglets decreased with increasing parity (P<0.001), by about two piglets from parity 1 to 5, whilst the percentage of weaned piglets was highest in parity 2 (86.9%) and then decreased thereafter (to 84.7% for parity 5) but this effect was more marginal (P=0.011).

Mortality increased with litter size (Table S4, Figure S3). SB% increased with TB (P<0.001), from about 1% for seven piglets to about 5% for 20 piglets. LBM% increased with BA (P<0.001) from about 6.5% for five piglets to 16.0% for 17 piglets, whilst TM% increased with TB (P<0.001) from about 8% for seven piglets to 23.5% for 20 piglets. The number weaned increased with LS_24h (P<0.001), but not with TB (P=0.874), however the percentage weaned decreased (P<0.001) with all measures of litter size, from about 93% for five BA piglets to 83% for 17 BA piglets. There was a positive correlation of TB with both total deaths (r_s_=0.425) and TM% (r_s_=0.414). Effects of pertinent litter size measures are similar (and all P<0.001 when tested last) in the GLMMs with all three fixed effects and the GLMMs with litter size alone.

Whilst effects of parity are also fairly similar, the model with all three fixed effects shows steady increases in mortality from parity 1 to 5 (all P<0.001 when tested last). Effects of pertinent litter size measures on LBM% are similar (P<0.001 when tested last) in the GLMM with all three fixed effects and the GLMM with litter size alone. Whilst effect of parity is also fairly similar, the model with all three fixed effects shows steady increases in crushing from parity 1 to 5 (P<0.001 when tested last – Table S5).

**Table S4.** Statistical tests from GLMMs for effects of parity and litter size on Key Performance Indicators, not adjusted for any other factors. All effects of litter size (TB, BA, LS_24h) are positive, except the shaded ones. Test statistics are (Wald/ndf) _ndf_ or F_ndf,ddf_.

|  | **Total mortality**  **(%)** | **Live-born mortality (%)** | **Stillborn (%)** | **Total born (TB)** | **Born alive (BA)** | **Number weaned** | **Weaned (%)** |
| --- | --- | --- | --- | --- | --- | --- | --- |
| **Parity** | 18.27_4_ | 2.80_4_ | 37.63_4_ | 24.22_4_ | 11.74_4_ | 26.25_4_ | 3.25_4_ |
|  | <0.001 | 0.024 | <0.001 | <0.001 | <0.001 | <0.001 | 0.011 |
| **Total born (TB)** | 338.16_1_ | 153.42_1_ | 116.27_1_ |  | 2105.13_1,3175_ | 2.25_1_ | 151.55_1_ |
|  | <0.001 | <0.001 | <0.001 |  | <0.001 | 0.134 | <0.001 |
| **Born alive (BA)** | 81.25_1_ | 138.98_1_ | 135.33_1_ | 2167.99_1,3175_ |  | 0.03 | 135.96_1_ |
|  | <0.001 | <0.001 | <0.001 | <0.001 |  | 0.874 | <0.001 |
| **Litter size at 24h (LS_24h)** | 1.08_1_ | 17.33_1_ | 11.41_1_ | 34.52_1_ | 49.52_1_ | 114.42_1_ | 29.78_1_ |
|  | 0.298 | <0.001 | <0.001 | <0.001 | <0.001 | <0.001 | <0.001 |

**Table S5**. Statistical tests from GLMMs for effects of system, parity and litter size on crushing and other types of mortality of all deaths (or deaths PRE and POST), not adjusted for any other factors. Shaded cells indicate dropped sow from random effects and results for Starved (%) by System are from LMM. Test statistics are (Wald/ndf)_ndf_ or F_ndf,ddf_.

|  | **Crushed**  **(%)** | **Low viability (%)** | **Starved (%)** | **Other (%)** | **Born dead (%)** | **PRE-Crushed (%)** | **POST-Crushed (%)** |
| --- | --- | --- | --- | --- | --- | --- | --- |
| **System** | 6.88_3,169_ | 0.80_3,178_ | 6.5_3,172_ | 4.73_3,157_ | 2.51_3,167_ | 1.26_3,124_ | 6.29_3,181_ |
|  | <0.001 | 0.496 | <0.001 | 0.003 | 0.060 | 0.291 | <0.001 |
| **Parity** | 1.57_4,2483_ | 26.91_4_ | 14.69_4_ | 12.97_4,2437_ | 34.64_4,2518_ | 2.83_4_ | 29.93_4_ |
|  | 0.180 | <0.001 | <0.001 | <0.001 | <0.001 | 0.023 | <0.001 |
| **TB** | 38.47_1_ | 1.98_1_ | 12.82_1_ | 3.82_1_ | 84.65_1_ | 3.68_1_ | 0.46_1_ |
|  | <0.001 | 0.159 | <0.001 | 0.051 | <0.001 | 0.055 | 0.497 |
| **BA** | 15.05_1,2437_ | 11.87_1_ | 3.86_1_ | 0.59_1_ | 50.74_1,2202_ | 1.00_1_ | 0.45_1_ |
|  | <0.001 | <0.001 | 0.050 | 0.442 | <0.001 | 0.317 | 0.501 |
| **LS_24h** | 18.13_1,1626_ | 309.19_1_ | 18.34_1_ | 2.54_1,1180_ | 41.88_1,1495_ | 0.31_1,1338_ | 164.64_1_ |
|  | <0.001 | <0.001 | <0.001 | 0.111 | <0.001 | 0.581 | <0.001 |

**Figures**


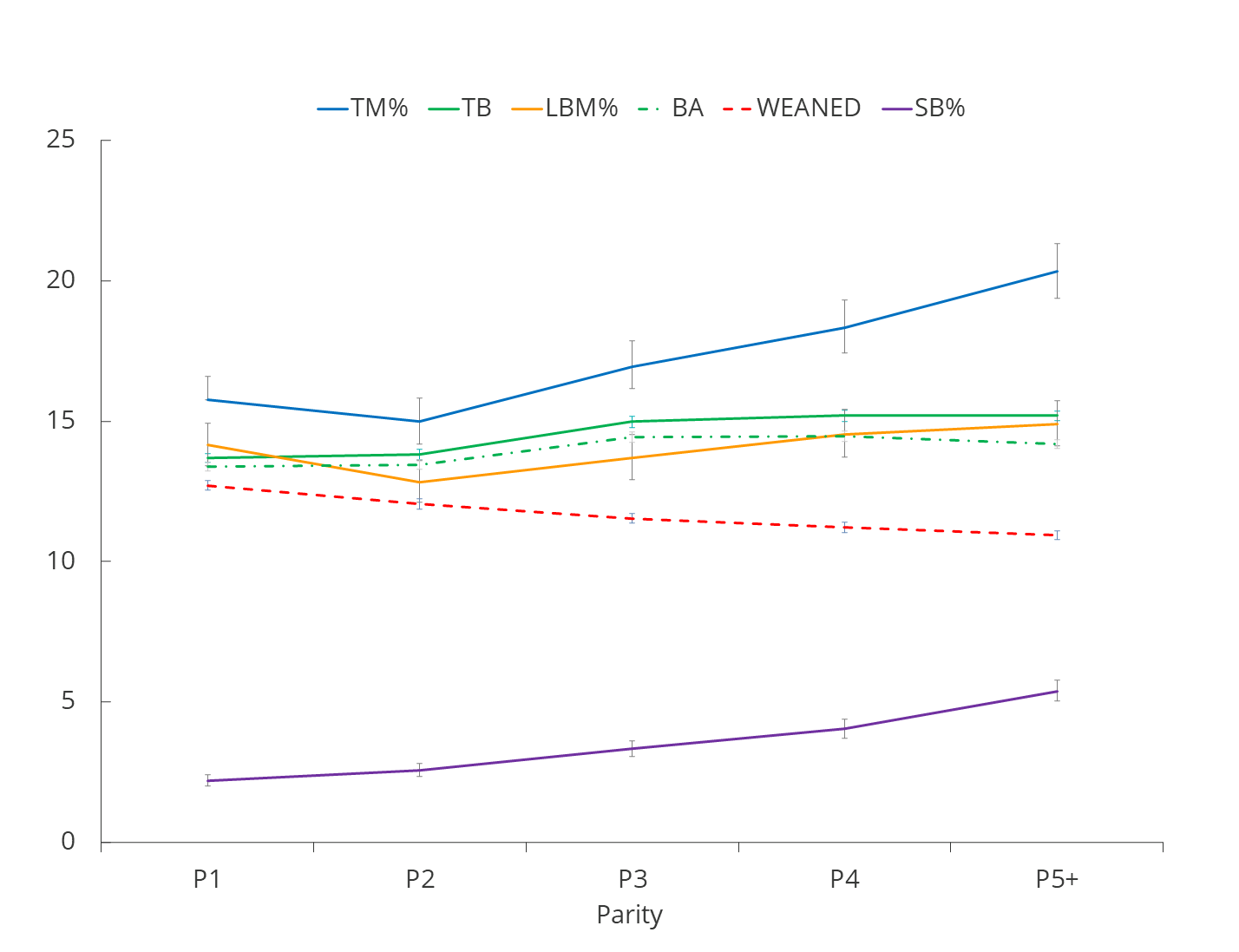


**Figure S2.** Effects of sow parity on Key Performance Indicators (TM%=Total Mortality%, LBM%=Live-born mortality%, SB%=Stillborn mortality%, TB=Total born, BA=Born alive). Lines show means from GLMMs and bars the means ± upper and lower standard errors (all back transformed). Parity 5+ combines the data for any sow of parity 5 or over.


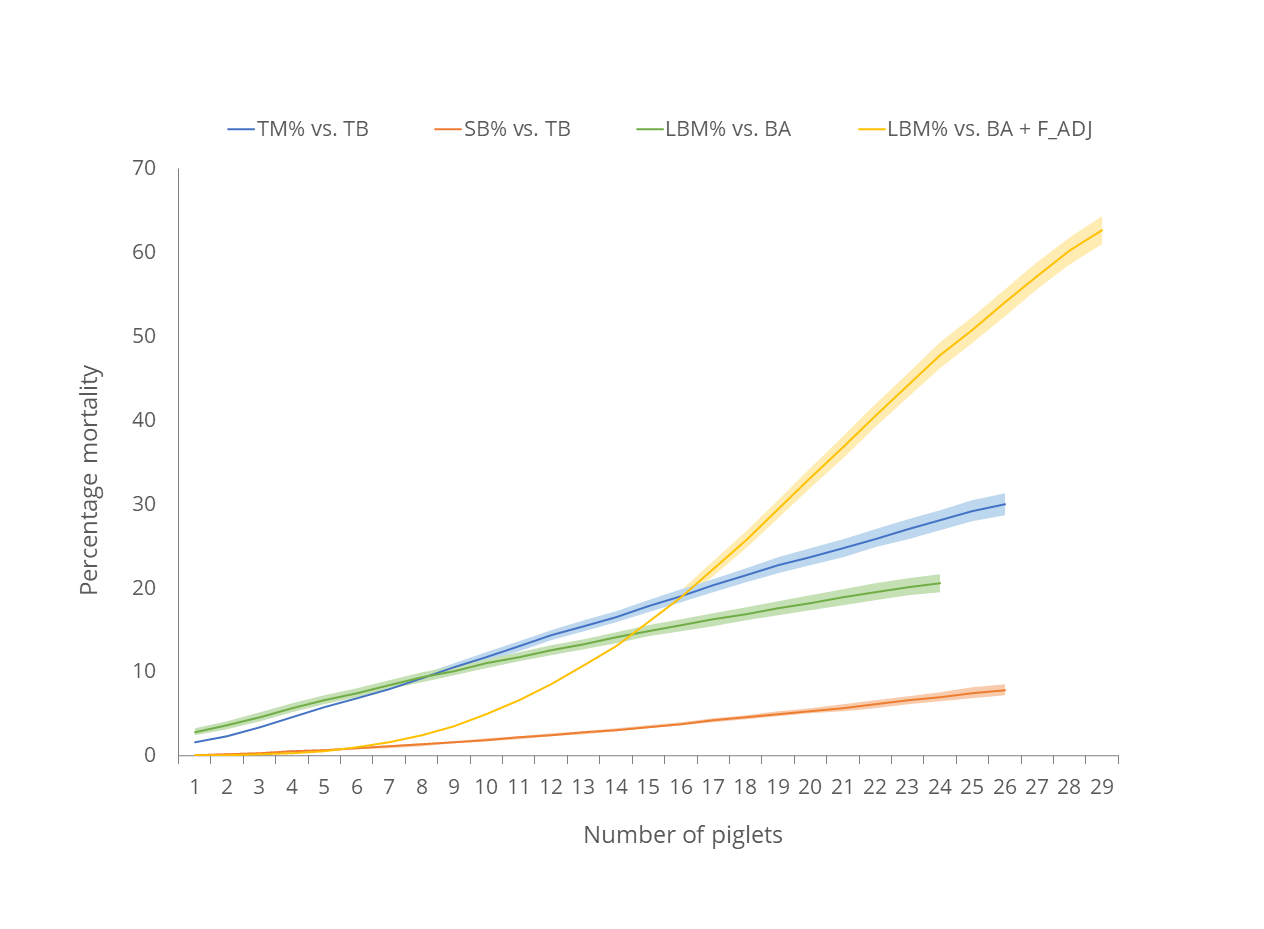


**Figure S3** – Effects of litter size variants TB=Total born; BA + F_ADJ = Born alive + fostering adjustment) on piglet mortality percentages (TM%=Total Mortality%; LBM%=Live-born mortality%; SB%=Stillborn mortality% - all P<0.001). Lines show means from GLMMs and shaded areas the means ± upper and lower standard errors (all back transformed).
